# ﻿Four new species of the genus *Tasactes* Faust, 1894 (Coleoptera, Curculionidae, Dryophthorinae) from China

**DOI:** 10.3897/zookeys.1256.160420

**Published:** 2025-10-17

**Authors:** Heyu Lü, Runzhi Zhang

**Affiliations:** 1 Key Laboratory of Animal Biodiversity Conservation and Integrated Pest Management, Chinese Academy of Sciences, No. 1 Beichen West Road, Chaoyang District, Beijing 100101, China Key Laboratory of Animal Biodiversity Conservation and Integrated Pest Management, Chinese Academy of Sciences Beijing China; 2 College of Life Science, University of Chinese Academy of Sciences, Beijing 100049, China University of Chinese Academy of Sciences Beijing China

**Keywords:** Diversity, morphology, Stromboscerini, taxonomy, weevils

## Abstract

Four new species of *Tasactes* Faust, 1894 are described from China: *Tasactes angustus***sp. nov.**, *Tasactes ocellatus***sp. nov.**, *Tasactes pilosus***sp. nov.** from Xizang Autonomous Region, and *Tasactes baoxingensis***sp. nov.** from Sichuan Province. Diagnostic characters are delineated through comparative morphology, supported by high-resolution habitus photographs and a key, with a distribution map of these species also provided.

## ﻿Introduction

The genus *Tasactes* Faust, 1894, a member of the tribe Stromboscerini (Curculionidae, Dryophthorinae), represents a taxonomically intricate and biogeographically significant group of forest-floor weevils. The keys to genera of recent Stromboscerini by Morimoto (1978, 1985) remain the definitive taxonomic framework for the tribe and serve as the primary reference for its systematics and assessment of diversity. *Tasactes* is diagnosed by its transversely truncated antennal club with a conically projecting velvety apex, 6-segmented funicle, and distinctly ventrally separated eyes. The genus *Tasactes* originally consisted of two species from Myanmar, *Tasactes carinulatus* Faust, 1894 and *Tasactes interruptus* Faust, 1894. The type species, *T. interruptus* was subsequently designated by Legalov (2021). However, Colonnelli (2024) was apparently was unaware of Legalov’s work and designated *T. carinulatus* as type species. The genus remained obscure for over a century, with no additional specimens reported until a recent molecular and morphological revision (Grebennikov 2018). Recent integrative studies have redefined *Tasactes* as a monophyletic genus nested within Asian Stromboscerini and characterized by two synapomorphies: a transversely truncate antennal club with conically projecting velvety apex (Grebennikov 2018). Molecular analyses (e.g. COI, ITS2, and 28S) further revealed eight allopatric and sympatric clades across southwestern China and Nepal, suggesting high cryptic diversity within the genus (Grebennikov 2018). Despite these advances, the genus remains understudied, with only three species formally described: *T. carinulatus*, *T. interruptus*, and *T. dudkoi* Legalov, 2021 (from Nepal).

In this study, we describe four new species from southwestern China, namely *Tasactes angustus* sp. nov., *Tasactes baoxingensis* sp. nov., *Tasactes ocellatus* sp. nov., and *Tasactes pilosus* sp. nov., These are the first formal descriptions of *Tasactes* species from China. While molecular data have proven valuable in delimiting clades in Stromboscerini, the present work prioritizes traditional taxonomic rigor, adhering to the International Code of Zoological Nomenclature (ICZN) and leveraging high-resolution imaging to document critical diagnostic features. By anchoring these taxa to morphologically defined species hypotheses, we aim to stabilize genus-level taxonomy and provide a foundation for future ecological or phylogenetic studies.

## ﻿Materials and methods

The specimens examined in this study were hand-collected, initially preserved in anhydrous ethanol, subsequently dried, and ultimately mounted on entomological pins using standard curatorial protocols. All specimens, including types, examined in this study, are deposited in the Institute of Zoology, Chinese Academy of Sciences, Beijing, China (**IZCAS**).

All morphological observations, measurements, and dissections were conducted using a Nikon SMZ 1500 stereomicroscope with coaxial LED illumination. Specimens imaged using a Canon EOS system, with detailed morphological features documented using a Canon 5D Mark II. Multi-focal image stacking was processed in Helicon Focus v. 7.5.4 Pro. All image plates were assembled and adjusted in Adobe Photoshop CC 2019. The species distribution map was generated in QGIS v. 3.28.15, integrating georeferenced locality data over a high-resolution basemap sourced from Tianditu (https://www.tianditu.gov.cn/, accessed 10.04.2025).

Measurements were taken and abbreviations are used as follows: body length (**Bl**) was measured from the anterior margin of the eye along the midline to the apex of the elytra. The length of the rostrum (**Rl**) was measured in lateral view from the apex to the anterior margin of the eye; the width of the rostrum (**Rw**) was measured at the widest point of the rostrum in dorsal view. The length of the pronotum (**Pl**) was measured along the midline from the apex to the base, whereas its width (**Pw**) was measured across at the widest point. The ratio of pronotum width to length was expressed as Pw/Pl. The length of the elytra (**El**) was measured along the midline from the transverse line joining the most anterior point of humeri to the apex whereas its width (**Ew** = body width (**Bw**)) was measured at the widest point. Proportions of the elytra were also expressed as a ratio El/Ew. The maximum width and length of ventrites and legs were taken as the greatest dimensions of each segment.

The Chinese common name is provided in Chinese characters, followed by its pinyin romanization in square brackets. Original label data have been written below in Chinese script. Added transliterations into pinyin or translations are placed between square brackets. Data from different labels are separated by two slashes (//) and lines within a label by one slash (/).

Terminology for general morphological structures primarily follows the online glossary of weevil characters proposed in the International Weevil Community Website (http://weevil.info/glossary-weevil-characters, accessed 10.04.2024). Male genitalia terminology follows Oberprieler et al. (2014), while terminology for certain leg structure follows Girón and Chamorro (2020).

## ﻿Taxonomic treatment

### ﻿Genus Tasactes

Taxon classificationAnimaliaColeopteraDryophthoridae

Faust, 1894

CBE03F43-FBF1-5625-B080-C21762290CB6

#### Type species.

*Tasactes interruptus* Faust, 1894.

#### Diagnosis.

(modified from Morimoto 1978, 1985). Body small; integument black, antennae and tarsomeres reddish-brown; head, rostrum, pronotum, abdominal ventrites densely covered with punctures; eyes linear or oval laterally, distinctly separated ventrally; antennal funicle 6-segmented, antennal club transversely truncated with conically projecting velvety apex; scutellum very small, usually subtriangular; elytra usually bearing pilose pustules, striae distinct; metepisternum entirely covered by elytra; legs usually covered with dense short pubescence; procoxae contiguous; femora unarmed; claws free; male genitalia usually without parameres, temones longer than pedon; lamina of female sternite 8 usually bifurcated; collum of spermatheca usually more developed than ramus.

### ﻿ Tasactes angustus

Taxon classificationAnimaliaColeopteraDryophthoridae

Lü & Zhang sp. nov.

54020A0B-B48C-5FFC-B9C9-19766D7B4B76

https://zoobank.org/304FA648-59A8-41F6-A9BA-716C832F18B1

**Figure 1.**
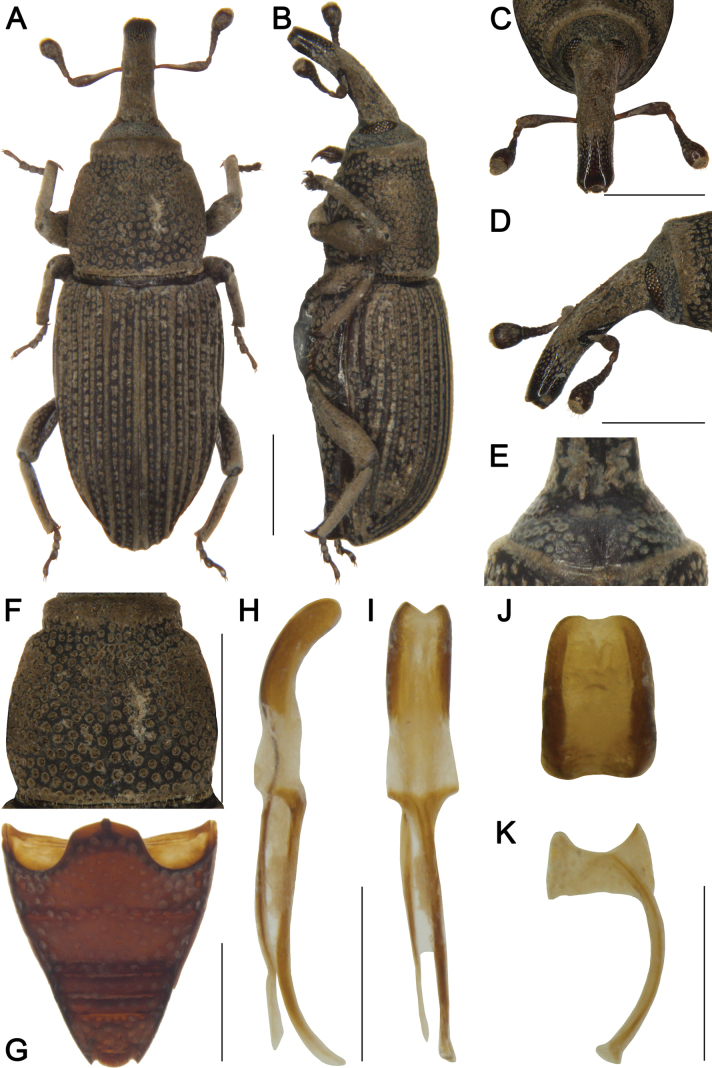
*Tasactes angustus* sp. nov., holotype male. A. Dorsal habitus; B. Lateral habitus; C. Head, dorsal view; D. Head, lateral view; E. Eyes, ventral view; F. Pronotum, dorsal view; G. Ventrites, ventral view; H. Penis, lateral view; I. Penis, ventral view; J. Penis at apex, showing details of the pedon; K. Spiculum gastrale. Scale bars: 1 mm (A–D, F, G); 0.5 mm (H, I, K).

**Figure 2.**
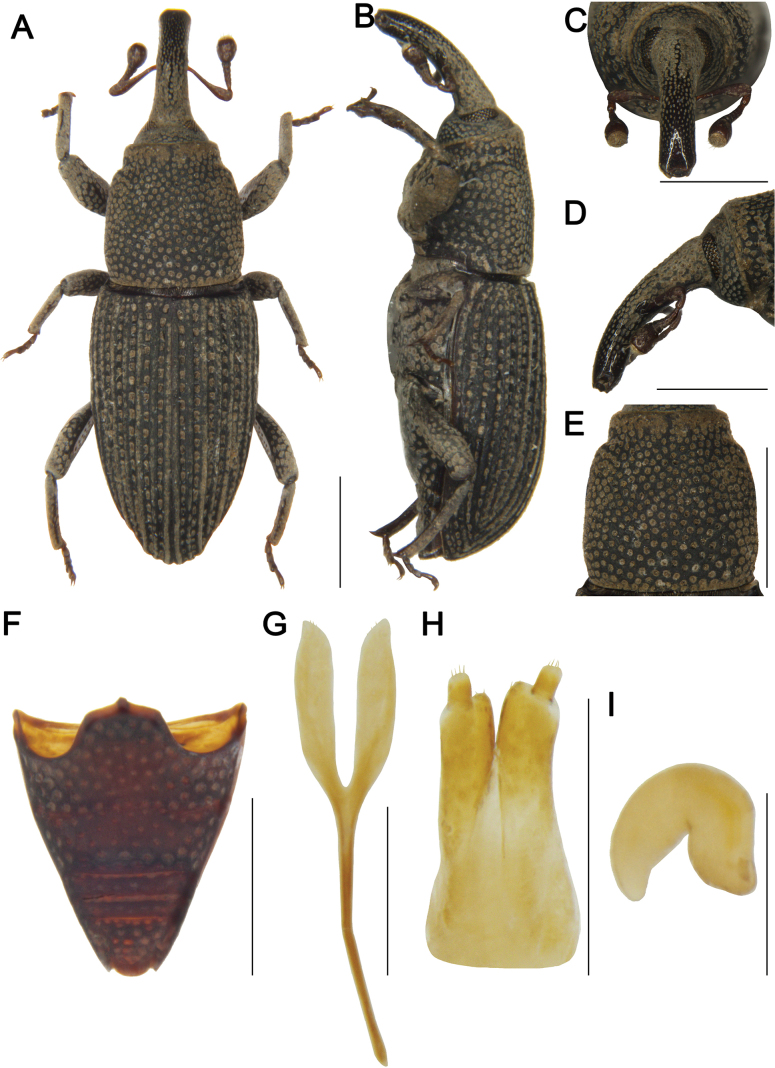
*Tasactes angustus* sp. nov., paratype female (IOZ(E)1965681). A. Dorsal habitus; B. Lateral habitus; C. Head, dorsal view; D. Head, lateral view; E. Pronotum, dorsal view; F. Ventrites, ventral view; G. Sternite 8; H. Ovipositor; I. Spermatheca. Scale bars: 1 mm (A–F); 0.25 mm (G–I).

Figs 1, 2 Chinese common name: 狭杯象 [Xiá bēi xiàng]

#### Material examined.

***Holotype***: ♂, labelled: printed: 西藏 波密县 易贡乡 S305省道11公里处 [Xīzàng, Bōmìxiàn, Yìgòngxiāng, S305 Shěngdào 11 Gōnglĭchù] / 30.1536°N, 94.9904°E / Alt. 2134 m / 2018.VIII.1 / 周润 [Zhōu Rùn] leg. // printed: IOZ(E)1965680. ***Paratypes***: 6♂6♀, same data as holotype, but printed label: IOZ(E)1965681–1965687, IOZ(E)1965689–1965692, IOZ(E)1965694; 2♂3♀, labelled: printed: 西藏 易贡 [Xīzàng, Yìgòng] / Alt. 2300 m / 1978.VII.13, 17 / 李法圣 [Lĭ Făshèng] leg. // printed: IOZ(E)1507485–1507488, IOZ(E)1507491; 1♂4♀, labelled: printed: 西藏 波密 易贡 [Xīzàng, Bōmì, Yìgòng] / Alt. 2300 m / 1987.VIII.17 / 韩寅恒 [Hán Yínhéng] leg. // printed: IOZ(E)1507489, IOZ(E)1507490, IOZ(E)1507492–1507494; 1♀, labelled: printed: 西藏 林芝市 波密县 易贡乡 [Xīzàng, Línzhīshì, Bōmìxiàn, Yìgòngxiāng] / 30.2398°N, 96.8624°E / Alt. 2289 m / 2019.VII.19 / 周润 [Zhōu Rùn] 马茁 [Mă Zhuó] leg. // printed: IOZ(E)1965693; 1♀, labelled: printed: 西藏 林芝市 波密县 易贡乡 [Xīzàng, Línzhīshì, Bōmìxiàn, Yìgòngxiāng] / K12 / 30.1506°N, 94.9952°E / Alt. 2097 m / 2019.VII.20 / 周润 [Zhōu Rùn] 马茁 [Mă Zhuó] leg. // printed: IOZ(E)1965688; 1♀, labelled: printed: 西藏 林芝市 墨脱县 墨脱公路 62k 喜荣沟附近 [Xīzàng, Línzhīshì, Mòtuōxiàn, MòtuōGōnglù, 62k, Xĭrónggōufùjìn] / 29.7110°N, 95.5881°E / Alt. 2749 m / 2019.VII.24 / 周润 [Zhōu Rùn] 马茁 [Mă Zhuó] leg. // printed: IOZ(E)1965689.

#### Type locality.

Yigong Township (11 kilometres from S305 provincial road), Bomi County, Linzhi City, Xizang Autonomous Region, China.

#### Comparative diagnosis.

*Tasactes angustus* sp. nov. morphologically is most similar to *T. carinulatus*, but distinguished by: (i) rostrum shorter than pronotum in both sexes, versus longer in *T. carinulatus*; (ii) pronotum sides rounded, lacking basal transverse depression, while sides subparallel with distinct basal transverse depression in *T. carinulatus*; (iii) elytral interstriae subequal in width and height, versus interstriae 1, 3, and 5 distinctly narrower and slightly lower in *T. carinulatus*; (iv) elytra more elongate with apical 1/6 conspicuously constricted, whereas lacking constriction in *T. carinulatus*.

#### Description.

(holotype, except female sternite 8 and genitalia).

***Coloration*** (Fig. 1A, B). Body entirely black; antennal scape and funicle, and tarsomeres reddish brown.

***Head*** (Fig. 1C–E). Forehead flat, slightly narrower than rostrum at base; eyes elongate-oval, distinctly separated ventrally; rostrum elongate (Rl/Rw 3.13), shorter than pronotum (Rl/Pl 0.85), slightly curved in lateral view, base thick, basal 1/3 with dense short, pubescence and coarse punctures; antennae inserted slightly anterior to middle of rostrum; scape long (l/w 4.42), not reaching eyes, gradually broadening from base to apex, apical 1/3 markedly widened; funicular segments 1 and 2 elongate, segment 2 funnel-shaped, segments 4–6 transverse; club subobconical (l/w 3.25).

***Pronotum*** (Fig. 1F). Longer than wide (Pl/Pw 1.04), widest posterior to middle, apical 1/6 distinctly constricted, sides rounded; disc slightly convex in lateral view, with dense, coarse punctures, punctures sparser on disc than laterally; densely covered with short pubescence; postocular lobes absent.

***Scutellum*.** Very small, elongate-oval.

***Elytra*.** Longer than wide (El/Ew 1.54), widest at basal 1/4, distinctly constricted at apical 1/6, sides rounded; disc nearly flat in lateral view; interstriae distinctly elevated, subequal in width, with dense, short pubescence; striae deep, punctures elongate-oval; distance between punctures subequal to a puncture length; punctures with dense short pubescence.

***Abdomen*** (Fig. 1G). Abdominal ventrites densely covered with coarse punctures; ventrite 2 with anterior margin slightly convex at middle, posterior margins of ventrites 2–4 rectilinear; ventrite 2 0.8 times length of ventrite 1, ventrite 3 slightly longer than ventrite 4, ventrite 5 1.9 times as wide as long.

***Legs*.** Densely covered with short pubescence; femora and tibiae with punctures; procoxae subconical, contiguous; profemur more robust than mesofemur and metafemur, femora unarmed; profemur 4.3 times as long as wide; tibiae bearing single long uncus; protibia 6.0 times as long as wide; tarsi long, tarsomeres 1–3 obconical, ventrally with dense erect setae, onychium elongate; claws free, divergent; protarsomere 1 1.7 times as long as wide, tarsomeres 2 and 3 each 1.3 times, onychium 4.0 times.

***Male genitalia*** (Fig. 1H–K). Pedon 0.4 times length of temones, evenly curved in lateral view, sides subparallel, base symmetrical, apex slightly narrowed; temones slender, slightly curved; manubrium of tegmen long, evenly curved from middle to apex, slightly wider than temones; spiculum gastrale robust, evenly curved; basal plate bifurcate, basal arms opposed, upper part of each basal arm approximately triangular, length subequal to width, apices with rounded angles.

***Female sternite 8 and genitalia*** (Fig. 2G–I). Sternite 8 with apodeme 1.5 times length of lamina; lamina bifurcate at base, sides slightly curved, apex with sparse setae; gonocoxites cylindrical, apices with dense setae; styli short, cylindrical, width approximately 1/2 width of gonocoxite apices, apices with setae; spermatheca with robust and curved cornu; corpus large; ramus and collum not developed.

#### Variation.

***Male paratypes*.** Measurements (in mm) (*n* = 9): Bl: 3.40–4.22 (3.87). Rl: 1.00–1.23 (1.16), Rw: 0.32–0.39 (0.36). Pl: 1.13–1.45 (1.30), Pw: 1.10–1.40 (1.26). El: 2.08–2.58 (2.40), Ew (Bw): 1.35–1.69 (1.55).

***Female paratypes*.** Measurements (in mm) (*n* = 16): Bl: 3.50–4.40 (3.94). Rl: 1.10–1.45 (1.27), Rw: 0.30–0.48 (0.39). Pl: 1.10–1.50 (1.32), Pw: 1.08–1.47 (1.27). El: 2.20–2.50 (2.43), Ew (Bw): 1.31–1.80 (1.53). Illustrations of a female are provided (Fig. 2A–I).

Male body slightly smaller than that of female; rostrum shorter in male; smooth from apical 1/3 to apex in male, smooth and shiny from middle to apex in female; antennae inserted slightly anterior to middle of rostrum in male, at middle in female; ventrites without distinct differences between sexes.

#### Distribution.

Bomi County and Medog County, Linzhi City, Xizang Autonomous Region, China (Fig. 9).

#### Etymology.

The species name is a Latin masculine adjective *angustus* (narrow), referring to the significantly narrower body width compared to other species within the genus.

### ﻿ Tasactes baoxingensis

Taxon classificationAnimaliaColeopteraDryophthoridae

Lü & Zhang sp. nov.

2212033A-64A0-5098-94D0-7E76BEF2C154

https://zoobank.org/3B9E2FA7-1098-4E91-99F8-902BB23225D2

**Figure 3.**
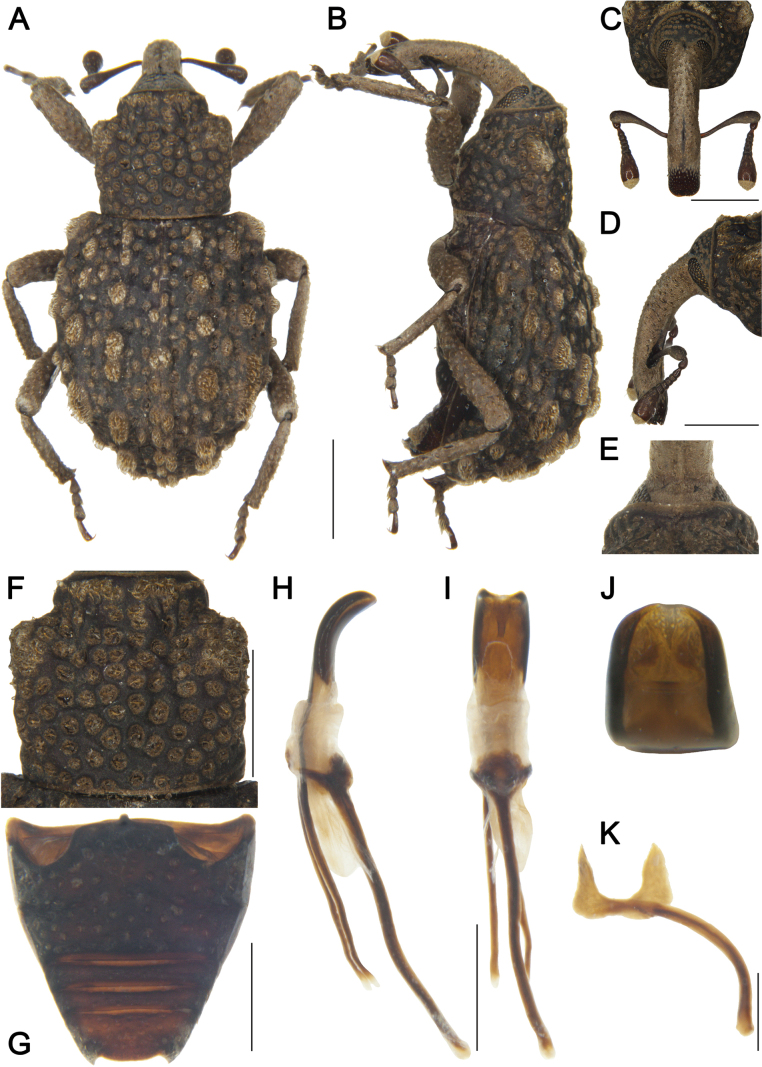
*Tasactes baoxingensis* sp. nov., holotype male. A. Dorsal habitus; B. Lateral habitus; C. Head, dorsal view; D. Head, lateral view; E. Eyes, ventral view; F. Pronotum, dorsal view; G. Ventrites, ventral view; H. Penis, lateral view; I. Penis, ventral view; J. Penis at apex, showing details of the pedon; K. Spiculum gastrale. Scale bars: 1 mm (A–D, F, G); 0.5 mm (H, I, K).

**Figure 4.**
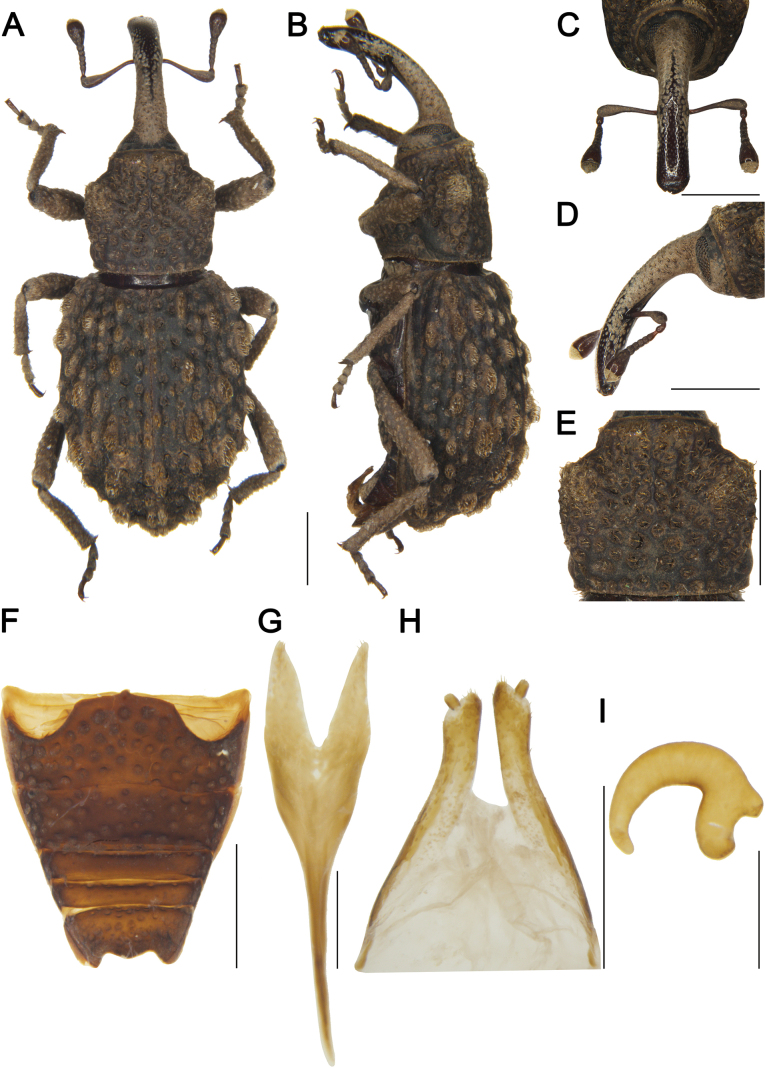
*Tasactes baoxingensis* sp. nov., paratype female (IOZ(E)1965701). A. Dorsal habitus; B. Lateral habitus; C. Head, dorsal view; D. Head, lateral view; E. Pronotum, dorsal view; F. Ventrites, ventral view; G. Sternite 8; H. Ovipositor; I. Spermatheca. Scale bars: 1 mm (A–F); 0.25 mm (G–I).

Figs 3, 4 Chinese common name: 宝兴杯象 [băo xīng bēi xiàng]

#### Material examined.

***Holotype***: ♂, labelled: printed: 四川 宝兴 锅巴岩 [Sìchuān, Băoxīng, Guōbāyán] / B17 / Alt. 3080 m / 杯诱 [bēiyòu] / 2001.VII.1–4 / 于晓东 [Yú xiăodōng] 周红章 [Zhōu hóngzhāng] leg. // printed: IOZ(E)1965700. ***Paratype***: 1♀, same data as holotype, except a printed label: IOZ(E)1965701.

#### Type locality.

Guoba Rock, Baoxing County, Yaan City, Sichuan Province, China.

#### Comparative diagnosis.

*Tasactes baoxingensis* sp. nov. resembles *T. dudkoi* but differs in the following characters: (i) body smaller (length 4.20–4.40 mm vs 5.40–6.20 mm in *T. dudkoi*); (ii) pronotum more distinctly constricted apically, lacking median carina (vs weak carina present in *T. dudkoi*), and postocular lobes absent (vs weakly developed in *T. dudkoi*); (iii) elytral pilose pustules distinctly wider than in *T. dudkoi* (see Legalov 2021: fig. 1); (iv) length ratio of pedon to temones exceeding that in *T. dudkoi* (see Legalov 2021: fig. 2).

#### Description.

(holotype, except female sternite 8 and genitalia).

***Coloration*** (Fig. 3A, B). Body entirely black; antennal scape and funicle, and tarsomeres reddish brown.

***Head*** (Fig. 3C–E). Forehead flat, slightly narrower than rostrum at base; eyes elongate-oval, distinctly separated ventrally; rostrum elongate (Rl/Rw 3.80), longer than pronotum (Rl/Pl 1.27), curved in lateral view, from base to apical 1/6 with short pubescence, apex with dense punctures; antennae inserted at apical 1/3 of rostrum; scape long (l/w 5.18), not reaching eyes, gradually widening from base to apex, apical 1/3 markedly widened; funicular segments 1 and 2 elongate, segment 1 1.3 times as long as segment 2, segment 2 funnel-shaped, segments 4–6 transverse; club subconical (l/w 2.00).

***Pronotum*** (Fig. 3F). Wider than long (Pl/Pw 0.97), widest at middle, apical 1/6 distinctly constricted, gradually narrowed from apical 1/4 to base; disc flat in lateral view, densely covered with coarse, deep punctures; distance between punctures much smaller than a puncture diameter; dense short pubescence present; postocular lobes absent.

***Scutellum*.** Small, subtriangular.

***Elytra*.** Longer than wide (El/Ew 1.16), widest at middle, sides rounded; disc nearly flat in lateral view; interstriae slightly convex, subequal in width, with dense short pubescence, odd interstriae bearing interrupted oval pilose pustules, pustules hemispherical in lateral view, width 2.0 times interstriae width; striae deep, punctures large, with dense pubescence; distance between punctures subequal to a puncture diameter.

***Abdomen*** (Fig. 3G). Abdominal ventrites densely covered with coarse punctures; ventrite 2 with anterior margin slightly convex at middle, posterior margins of ventrites 2–4 rectilinear; ventrite 2 0.9 times length of ventrite 1, ventrite 3 slightly longer than ventrite 4, ventrite 5 2.3 times as wide as long.

***Legs*.** Densely covered with short pubescence; femora and tibiae with punctures; procoxae subconical, contiguous; profemur more robust than mesofemur and metafemur, femora unarmed; profemur 3.8 times as long as wide; tibiae bearing single long uncus; protibia 5.1 times as long as wide; tarsi long, tarsomeres 1–3 obconical, ventrally with setae, onychium elongate; claws free, divergent; protarsomere 1 1.7 times as long as wide, tarsomere 2 and 3 each 1.3 times, onychium 3.5 times.

***Male genitalia*** (Fig. 3H–K). Pedon 0.3 times longer than temones, curved in lateral view, sides subparallel, base symmetrical, apex slightly narrowed; temones slender, slightly curved; manubrium of tegmen robust, elongate, slightly curved, approximately 3.0 times as wide as temones; spiculum gastrale robust, evenly curved; basal plate bifurcate, basal arms opposed, upper part of each basal arm approximately triangular, length subequal to width, apices with rounded angles.

***Female sternite 8 and genitalia*** (Fig. 4G–I). Sternite 8 with apodeme length subequal to that of lamina; lamina bifurcate at middle, sides curved, apex with sparse setae; gonocoxites cylindrical, apices with dense setae; styli short, cylindrical, width approximately 1/3 width of gonocoxite apices, apices with short setae; spermatheca with curved, apically rounded cornu; corpus robust; ramus longer than collum.

#### Variation.

***Male holotype*.** Measurements (in mm): Bl: 4.52. Rl: 1.90, Rw: 0.50. Pl: 1.50, Pw: 1.55. El: 2.90, Ew (Bw): 2.50.

***Female paratype*.** Measurements (in mm): Bl: 5.38. Rl: 2.00, Rw: 0.50. Pl: 1.80, Pw: 1.90. El: 2.61, Ew (Bw): 2.70. Illustrations of a female are provided (Fig. 4A–I).

Female rostrum smooth and shiny from middle to apex, while male rostrum smooth only at apex; female antennae inserted at middle of rostrum, whereas male antennae inserted at apical 1/3.

#### Distribution.

Known only from the type locality in Sichuan Province, China (Fig. 9).

#### Etymology.

This species is named after its type locality, Baoxing County. Adjective, variable.

### ﻿ Tasactes ocellatus

Taxon classificationAnimaliaColeopteraDryophthoridae

Lü & Zhang sp. nov.

2F69D9C7-D7D9-57FE-A5FF-3B46EA33E484

https://zoobank.org/C75098A1-7538-4938-ACC8-FED7AE57EF76

**Figure 5.**
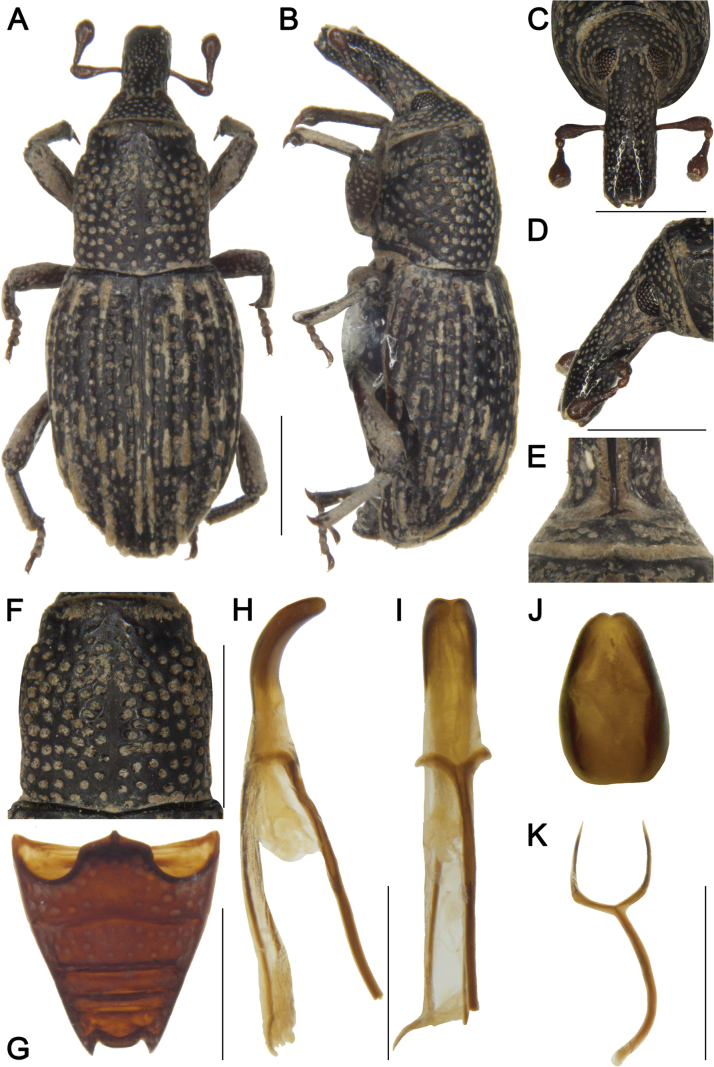
*Tasactes ocellatus* sp. nov., holotype male. A. Habitus dorsal; B. Lateral habitus; C. Head, dorsal view; D. Head, lateral view; E. Eyes, ventral view; F. Pronotum, dorsal view; G. Ventrites, ventral view; H. Penis, lateral view; I. Penis, ventral view; J. Penis at apex, showing details of the pedon; K. Spiculum gastrale. Scale bars: 1 mm (A–D, F, G); 0.5 mm (H, I, K).

**Figure 6.**
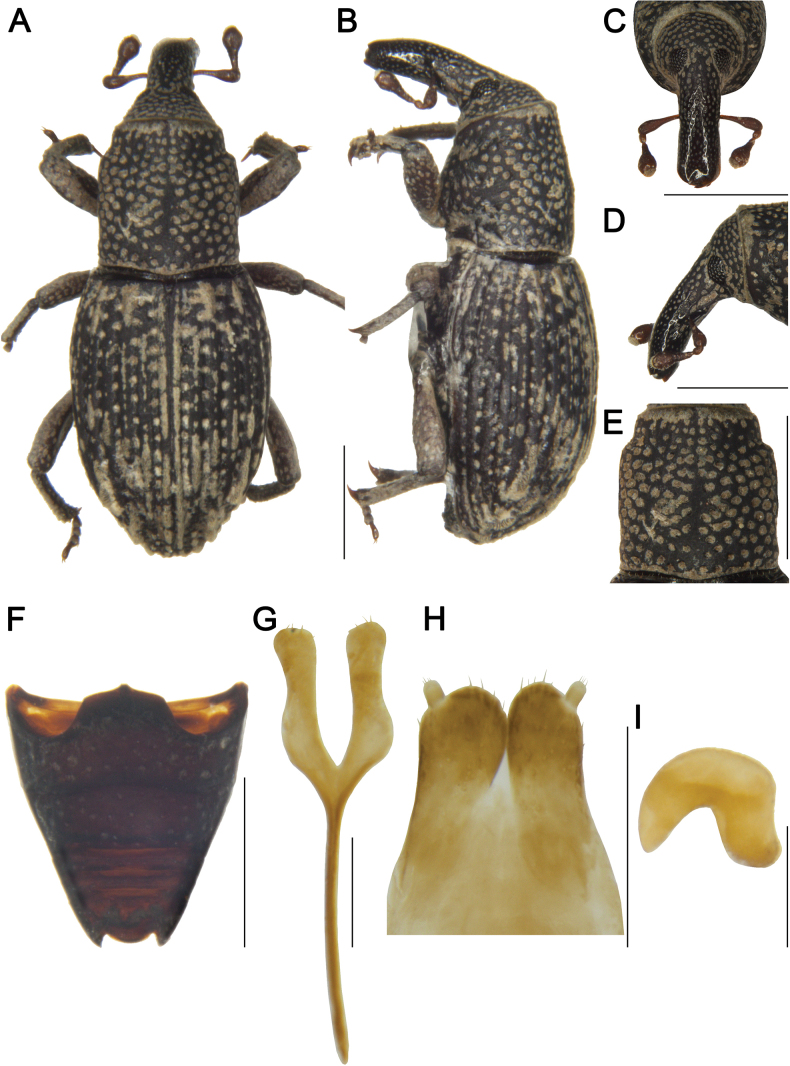
*Tasactes ocellatus* sp. nov., paratype female (IOZ(E)1965696). A. Dorsal habitus; B. Lateral habitus; C. Head, dorsal view; D. Head, lateral view; E. Pronotum, dorsal view; F. Ventrites, ventral view; G. Sternite 8; H. Ovipositor; I. Spermatheca. Scale bars: 1 mm (A–F); 0.25 mm (G–I).

Figs 5, 6 Chinese common name: 小眼杯象 [xiăo yăn bēi xiàng]

#### Material examined.

***Holotype***: ♂, labelled: printed: 西藏 林芝市 墨脱县 背崩乡 阿苍村 [Xīzàng, Línzhīshì, Mòtuōxiàn, Bèibēngxiāng, Acāngcūn] / 2019.VIII.09 // 29.2453°N, 95.1300°E / Alt. 1409 m / 周润 [Zhōu Rùn] 马茁 [Mă Zhuó] leg. // printed: IOZ(E)1965695. ***Paratypes***: 2♀, same data as holotype, but printed label: IOZ(E)1965696 and IOZ(E)1965697 respectively.

#### Type locality.

Acang Village, Beibeng Township, Medog County, Linzhi City, Xizang Autonomous Region, China.

#### Comparative diagnosis.

*Tasactes ocellatus* sp. nov. is most similar to *T. carinulatus* but differs in the following characters: (i) body smaller (length 3.65–3.89 mm, width 1.66–1.68 mm vs length 4.20 mm, width 1.80 mm in *T. carinulatus*); (ii) rostrum shorter than pronotum in both sexes, whereas longer than pronotum in *T. carinulatus*; (iii) pronotum lacking basal transverse depression, while distinct basal transverse depression present in *T. carinulatus*; (iv) elytral punctures subequal in size to pronotal punctures, while 2.0 times as large as pronotal punctures in *T. carinulatus*. *Tasactes ocellatus* sp. nov. geographically closest to *T. angustus* sp. nov., both occurring in Medog County, but differing in: (i) eyes barely visible ventrally, versus distinctly visible in *T. angustus* sp. nov.; (ii) rostrum shorter (Rl 0.95–1.05 mm vs Rl 1.00–1.45 mm in *T. angustus* sp. nov.); (iii) antennal scape shorter and stouter (l/w 3.78 vs l/w 4.42 in *T. angustus* sp. nov.); (iv) pronotum sides subparallel, versus sides curved in *T. angustus* sp. nov.; (v) elytral interstriae 1–3 flat, bearing pilose pustules, whereas uniformly convex, lacking pustules in *T. angustus* sp. nov.; (vi) pedon apex acutely narrowed, manubrium shorter than temones, while apex slightly narrowed, manubrium subequal to temones in *T. angustus* sp. nov.; (vii) basal arms of spiculum gastrale slender and linear, as compared to subtriangular in *T. angustus* sp. nov.; (viii) styli width approximately 1/4 width of gonocoxite apices, while 1/2 in *T. angustus* sp. nov.

#### Description.

(holotype, except female sternite 8 and genitalia).

***Coloration*** (Fig. 5A, B). Body entirely black; antennal scape and funicle, and tarsomeres reddish brown.

***Head*** (Fig. 5C–E). Forehead flat, 0.5 times width of rostrum at base; eyes small, oval, barely visible ventrally; rostrum elongate (Rl/Rw 1.96), shorter than pronotum (Rl/Pl 0.73), slightly curved in lateral view, base thick, densely covered with punctures, bearing short pubescence; antennae inserted slightly anterior to middle of rostrum; scape long (l/w 3.78), not reaching eyes, gradually widening from base to apex, middle markedly widened; funicular segments 1 and 2 elongate, segment 2 funnel-shaped, segments 4–6 transverse; club subobconical (l/w 1.64).

***Pronotum*** (Fig. 5F). Longer than wide (Pl/Pw 1.18), widest at middle, apical 1/5 distinctly constricted, sides subparallel; disc slightly convex in lateral view, with dense and coarse punctures; distance between punctures slightly exceeding puncture diameter, punctures sparser on disc than laterally; densely covered with short pubescence; postocular lobes absent.

***Scutellum*.** Very small, subtriangular.

***Elytra*.** Longer than wide (El/Ew 1.44), widest at basal 1/4, sides rounded; disc slightly convex in lateral view; interstriae slightly convex, subequal in width, interstriae 3 and 5 basally, interstriae 1–5 from middle to apex bearing pilose pustules forming indistinct V-shaped pattern; striae deep, punctures rounded, bearing dense, short pubescence; distance between punctures slightly exceeding puncture diameter.

***Abdomen*** (Fig. 5G). Abdominal ventrites densely covered with coarse punctures; ventrite 2 with anterior margin slightly convex at middle, posterior margins of ventrites 2–4 rectilinear; ventrite 2 0.6 times length of ventrite 1, ventrite 3 slightly longer than ventrite 4, ventrite 5 2.3 times as wide as long.

***Legs*.** Densely covered with short pubescence; femora and tibiae with punctures; procoxae subconical, contiguous; profemur more robust than mesofemur and metafemur, femora unarmed; profemur 3.9 times as long as wide; tibiae bearing single long uncus; protibia 5.7 times as long as wide; tarsi long, tarsomeres 1–3 obconical, ventrally with erect setae, onychium elongate; claws free, divergent; protarsomere 1 1.7 times as long as wide, tarsomeres 2 and 3 each 1.1 times, onychium 4.2 times.

***Male genitalia*** (Fig. 5H–K). Pedon 0.3 times length of temones, evenly curved in lateral view, sides subparallel, base symmetrical, apex distinctly narrowed; temones slender, slightly curved; manubrium of tegmen long, 0.7 times length of temones, slightly curved, wider than temones; spiculum gastrale robust, evenly curved; basal plate bifurcate, basal arms slender, opposed, apices acute.

***Female sternite 8 and genitalia*** (Fig. 6G–I). Sternite 8 with apodeme 1.5 times length of lamina; lamina bifurcate at base, sides curved and depressed, apex with sparse setae; gonocoxites cylindrical, apices with dense setae; styli short, cylindrical, width approximately 1/4 width of gonocoxite apices, apices with short setae; spermatheca with robust and curved cornu; corpus large; ramus and collum not developed.

#### Variation.

***Male holotype*.** Measurements (in mm): Bl: 3.89. Rl: 0.98, Rw: 0.50. Pl: 1.33, Pw: 1.13. El: 2.38, Ew (Bw): 1.68.

***Female paratypes*.** Measurements (in mm) (*n* = 2): Bl: 3.65–3.85 (3.75). Rl: 0.95–1.05 (1.00), Rw: 0.39–0.40 (0.40). Pl: 1.24–1.3 (1.27), Pw: 1.09–1.01 (1.10). El: 2.10–2.35 (2.23), Ew (Bw): 1.55–1.65 (1.60). Illustrations of a female are provided (Fig. 6A–I).

Female rostrum slenderer than that of male; male antennae inserted slightly anterior to middle of rostrum, female antennae inserted at middle of rostrum; ventrites without distinct differences between sexes.

#### Distribution.

Known only from the type locality in Xizang, China (Fig. 9).

#### Etymology.

The species name is a Latin masculine adjective *ocellatus* (having small eyes), referring to the significantly reduced eyes compared to other species within the genus.

### ﻿ Tasactes pilosus

Taxon classificationAnimaliaColeopteraDryophthoridae

Lü & Zhang sp. nov.

6C39C57C-4AB2-561E-9F06-221D8C2112EB

https://zoobank.org/137F5BB7-5EC2-40AE-9306-97AF63D818CA

**Figure 7.**
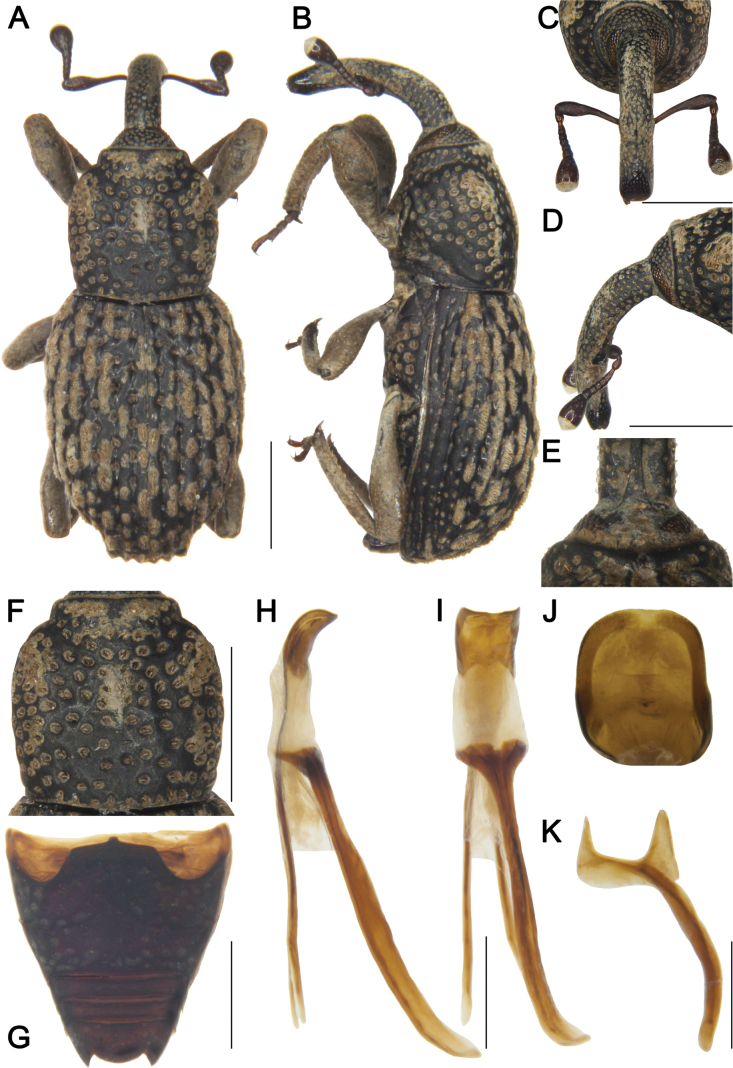
*Tasactes pilosus* sp. nov., holotype male. A. Dorsal habitus; B. Lateral habitus; C. Head, dorsal view; D. Head, lateral view; E. Eyes, ventral view; F. Pronotum, dorsal view; G. Ventrites, ventral view; H. Penis, lateral view; I. Penis, ventral view; J. Penis at apex, showing details of the pedon; K. Spiculum gastrale. Scale bars: 1 mm (A–D, F, G); 0.5 mm (H, I, K).

**Figure 8.**
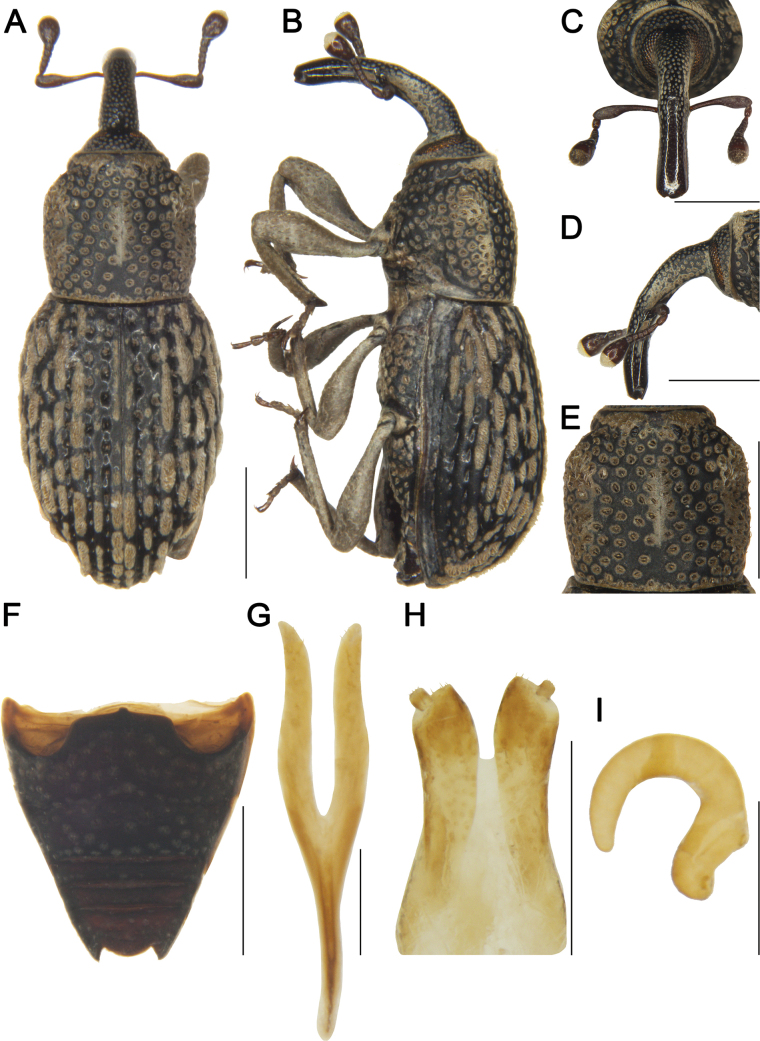
*Tasactes pilosus* sp. nov., paratype female (IOZ(E)1965699). A. Dorsal habitus; B. Lateral habitus; C. Head, dorsal view; D. Head, lateral view; E. Pronotum, dorsal view; F. Ventrites, ventral view; G. Sternite 8; H. Ovipositor; I. Spermatheca. Scale bars: 1 mm (A–F); 0.25 mm (G–I).

Figs 7, 8 Chinese common name: 毛杯象 [máo bēi xiàng]

#### Material examined.

***Holotype***: ♂, labelled: printed: 西藏 林芝市 巴宜区 排龙乡 迫龙沟 [Xīzàng, Línzhīshì, Bāyíqū, Páilóngxiāng, Pòlónggōu] / D2 / 30.02421°N, 95.00770°E / Alt. 1923 m / 2018.VIII.3 / 周润 [Zhōu Rùn] leg. // printed: IOZ(E)1965698. ***Paratype***: 1♀, same data as holotype, except a printed label: IOZ(E)1965699.

#### Type locality.

Polong Gully, Pailong Township, Bayi District, Linzhi City, Xizang Autonomous Region, China.

#### Comparative diagnosis.

*Tasactes pilosus* sp. nov. resembles *T. dudkoi* but differs unequivocally in: (i) body smaller (length 4.20–4.40 mm vs 5.40–6.20 mm in *T. dudkoi*); (ii) antennal scape shorter and stouter (l/w 4.75 vs l/w 6.70 in *T. dudkoi*); (iii) pronotum lacking median carina, whereas with weak median carina in *T. dudkoi*; (iv) pronotum bearing median longitudinal pilose pustule, while the latter is absent in *T. dudkoi* (see Legalov 2021: fig. 1); (v) temones distinctly more slender than in *T. dudkoi* (see Legalov 2021: fig. 2).

#### Description.

(holotype, except female sternite 8 and genitalia).

***Coloration*** (Fig. 7A, B). Body entirely black; antennal scape and funicle, and tarsomeres reddish brown.

***Head*** (Fig. 7C–E). Forehead flat, slightly narrower than rostrum base width; eyes elongate-oval, distinctly separated ventrally; rostrum elongate (Rl/Rw 5.16), longer than pronotum (Rl/Pl 1.10), evenly curved in lateral view, from base to apical 1/6 with short pubescence, apex with dense punctures; antennae inserted at apical 1/3 of rostrum; scape long (l/w 4.75), not reaching eyes, gradually widening from base to apex, apical 1/3 markedly widened; funicular segments 1 and 2 elongate, segment 2 funnel-shaped, segments 4–6 transverse; club subobconical (l/w 1.66).

***Pronotum*** (Fig. 7F). Longer than wide (Pl/Pw 1.07), widest at middle, apical 1/8 distinctly constricted, sides rounded; disc slightly convex in lateral view, densely covered with coarse punctures, punctures sparser on disc than laterally; dense, short pubescence forming median longitudinal pilose pustule; postocular lobes weak.

***Scutellum*.** Small, subtriangular.

***Elytra*.** Longer than wide (El/Ew 1.27), widest at basal 1/4, apical 1/6 distinctly constricted, sides rounded; disc nearly flat in lateral view; interstriae convex, subequal in width, with dense short pubescence, interstriae 1–8 bearing interrupted longitudinal pilose pustules, pustule width subequal to interstrial width; striae deep, punctures large, bearing dense, short pubescence; distance between punctures exceeding puncture diameter.

***Abdomen*** (Fig. 7G). Abdominal ventrites densely covered with coarse punctures; ventrite 2 with anterior margin slightly convex at middle, posterior margins of ventrites 2–4 rectilinear; ventrite 2 0.8 times length of ventrite 1, ventrite 3 slightly longer than ventrite 4, ventrite 5 2.0 times as wide as long.

***Legs***. Densely covered with short pubescence; femora and tibiae with punctures; procoxae subconical, contiguous; profemur more robust than mesofemur and metafemur, femora unarmed; profemur 3.3 times as long as wide; tibiae bearing single long uncus; protibia 6.2 times as long as wide; tarsi long, tarsomeres 1–3 obconical, ventrally with setae, onychium elongate; claws free, divergent; protarsomere 1 1.8 times as long as wide, tarsomere 2 1.3 times, tarsomere 3 1.4 times, onychium 4.3 times.

***Male genitalia*** (Fig. 7H–K). Pedon 0.2 times length of temones, curved in lateral view, sides curved, base symmetrical, apex broad and not narrowed; temones slender, slightly curved; manubrium of tegmen very robust, elongate, apex curved, approximately 3.0 times as wide as temones; spiculum gastrale robust, evenly curved; basal plate bifurcate, basal arms opposed, upper part of each basal arm approximately triangular, length subequal to width, apices with rounded angles.

***Female sternite 8 and genitalia*** (Fig. 8G–I). Sternite 8 with apodeme 0.8 times length of lamina; lamina bifurcate at base, sides curved, apex with sparse setae; gonocoxites cylindrical, apices with dense setae; styli short, cylindrical, width approximately 1/4 width of gonocoxite apices, apices with short setae; spermatheca with curved, apically rounded cornu; corpus robust; ramus and collum weakly developed.

#### Variation.

***Male holotype*.** Measurements (in mm): Bl: 4.20. Rl: 1.65, Rw: 0.32. Pl: 1.50, Pw: 1.40. El: 2.42, Ew (Bw): 1.90.

***Female paratype*.** Measurements (in mm): Bl: 4.40. Rl: 1.60, Rw: 0.35. Pl: 1.51, Pw: 1.45. El: 2.61, Ew (Bw): 1.98. Illustrations of a female are provided (Fig. 8A–I).

Female rostrum smooth and shiny from middle to apex, while male rostrum smooth only at apex; female antennae inserted at middle of rostrum, whereas male antennae inserted at apical 1/3.

#### Distribution.

**Figure 9.**
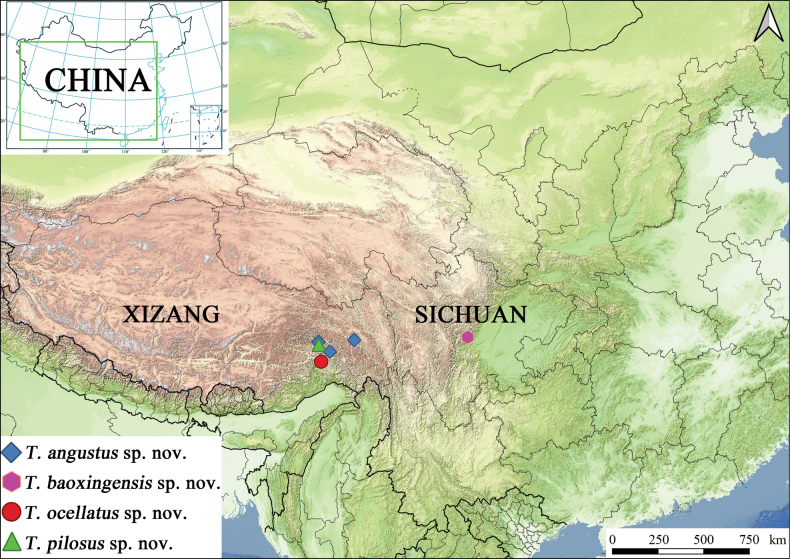
Distribution of *Tasactes* species in China (based on specimens examined in this study).

Known only from the type locality in Xizang, China (Fig. 9).

#### Etymology.

The specific name is derived from the Latin word *pilosus* (hairy), referring to the dense pubescence on the pronotum, particularly the distinct longitudinal tuft of pubescence medially. Adjective, variable.

##### ﻿Key to species of the genus *Tasactes*

**Table:** 

1	Elytra without interrupted pilose pustules	**2**
–	Elytra with interrupted pilose pustules	**3**
2	Rostrum shorter than pronotum. Pronotal sides rounded, lacking basal transverse depression. Elytral interstriae subequal in width and height. Body length 3.40–4.40 mm, width 1.31–1.80 mm	***T. angustus* sp. nov.**
–	Rostrum longer than pronotum. Pronotal sides subparallel, with basal transverse depression. Elytral interstriae 1, 3 and 5 distinctly narrower than others. Body length 4.20 mm, width 1.80 mm	***T. carinulatus***
3	Pronotum punctate	**4**
–	Pronotum rugose-punctate. Body length 4.00–4.80 mm, width 1.81–2.20 mm	***T. interruptus***
4	Pronotum with weak postocular lobes	**5**
–	Pronotal postocular lobes absent	**6**
5	Antennal scape L/W ratio 6.70. Pronotum with weak median carina, lacking median longitudinal pilose pustule. Body length 5.40–6.20 mm	***T. dudkoi***
–	Antennal scape L/W ratio 4.75. Pronotum without median carina, bearing median longitudinal pilose pustule. Body length 4.20–4.40 mm, width 1.91–1.98 mm	***T. pilosus* sp. nov.**
6	Eyes distinctly visible in ventral view, laterally linear (Figs 3D, 4D). Rostrum longer than pronotum. Antennal scape L/W ratio 5.18. Pronotum wider than long. Elytral pustules hemispherical in lateral view. Body length 4.20–4.40 mm, width 2.50–2.70 mm	***T. baoxingensis* sp. nov.**
–	Eyes barely visible in ventral view, laterally oval (Figs 5D, 6D). Rostrum shorter than pronotum. Antennal scape L/W ratio 3.78. Pronotum longer than wide. Elytral pustules linear in lateral view. Body length 3.65–3.89 mm, width 1.66–1.68 mm	***T. ocellatus* sp. nov.**

## ﻿Discussion

The four new Chinese species described here conform to criteria diagnostic of *Tasactes*, reinforcing the morphological coherence of this genus. Of these new species, *Tasactes baoxingensis* sp. nov. is characterized by having prominent hemispherical pilose pustules on the elytra, which are uncommon in the genus. However, image data provided by Grebennikov (2018) indicate that elytral sculpture shape varies considerably among *Tasactes* species. Furthermore, phylogenetic analyses show that even closely related species can significantly differ in body size and the shape of both the pronotum and elytra. Therefore, we confirm that *T. baoxingensis* sp. nov. can be placed in the genus *Tasactes*. Significantly, specimens with distinct morphological divergence were found during examination, including taxa bearing two unci on the protibiae and mesotibiae and sharp tubercles at apical 1/4 of the side of the pronotum, features not found in all previously known species (including the new species). Though these morphotypes likely represent novel evolutionary lineages, their formal taxonomic treatment remains pending due to specimen preservation issues (partial structural loss) and representation by only one sex.

These findings align with Grebennikov’s (2018) predictions that diversity of *Tasactes* in southwestern China, particularly in Sichuan, Xizang, Yunnan, and Taiwan, is greater than currently known; unique habitats likely harbor numerous undescribed cryptic species. Taxonomic studies on Chinese Dryophthorinae by He (2001, unpublished Master’s thesis) further corroborate this pattern.

Considering the small size of these taxa and the limited ecological information currently available, we propose implementing systematic forest-floor litter sifting for targeted sampling. Integration of morphological dissection, molecular phylogenetics and host plant association data will elucidate intergeneric relationships within the Stromboscerini.

## Supplementary Material

XML Treatment for Tasactes

XML Treatment for Tasactes angustus

XML Treatment for Tasactes baoxingensis

XML Treatment for Tasactes ocellatus

XML Treatment for Tasactes pilosus

## ﻿Additional information

### Use of AI

No use of AI was reported.

### Author ORCIDs

Heyu Lü https://orcid.org/0000-0002-9719-3966

Runzhi Zhang https://orcid.org/0000-0001-9001-0154

### Data availability

All of the data that support the findings of this study are available in the main text.
